# Development and validation of mortality prediction models for heart transplantation using nutrition-related indicators: a single-center study from China

**DOI:** 10.3389/fcvm.2024.1346202

**Published:** 2024-02-26

**Authors:** Shirui Qian, Bingxin Cao, Ping Li, Nianguo Dong

**Affiliations:** ^1^ Department of Cardiovascular Surgery, Union Hospital, Tongji Medical College, Huazhong University of Science and Technology, Wuhan, China; ^2^ Key Laboratory of Organ Transplantation, Ministry of Education NHC, Chinese Academy of Medical Sciences, Wuhan, China

**Keywords:** heart transplantation, survival, prediction model, risk stratification, heart failure

## Abstract

**Objective:**

We sought to develop and validate a mortality prediction model for heart transplantation (HT) using nutrition-related indicators, which clinicians could use to identify patients at high risk of death after HT.

**Method:**

The model was developed for and validated in adult participants in China who received HT between 1 January 2015 and 31 December 2020. 428 subjects were enrolled in the study and randomly divided into derivation and validation cohorts at a ratio of 7:3. The likelihood-ratio test based on Akaike information was used to select indicators and develop the prediction model. The performance of models was assessed and validated by area under the curve (AUC), C-index, calibration curves, net reclassification index, and integrated discrimination improvement.

**Result:**

The mean (SD) age was 48.67 (12.33) years and mean (SD) nutritional risk index (NRI) was 100.47 (11.89) in the derivation cohort. Mortality after HT developed in 66 of 299 patients in the derivation cohort and 28 of 129 in the validation cohort. Age, NRI, serum creatine, and triglyceride were included in the full model. The AUC of this model was 0.76 and the C statistics was 0.72 (95% CI, 0.67–0.78) in the derivation cohort and 0.71 (95% CI, 0.62–0.81) in the validation cohort. The multivariable model improved integrated discrimination compared with the reduced model that included age and NRI (6.9%; 95% CI, 1.8%–15.1%) and the model which only included variable NRI (14.7%; 95% CI, 7.4%–26.2%) in the derivation cohort. Compared with the model that only included variable NRI, the full model improved categorical net reclassification index both in the derivation cohort (41.8%; 95% CI, 9.9%–58.8%) and validation cohort (60.7%; 95% CI, 9.0%–100.5%).

**Conclusion:**

The proposed model was able to predict mortality after HT and estimate individualized risk of postoperative death. Clinicians could use this model to identify patients at high risk of postoperative death before HT surgery, which would help with targeted preventative therapy to reduce the mortality risk.

## 1 Introduction

Heart failure (HF) is a global pandemic. There are about 64.3 million HF patients worldwide and approximately 4.5 million in China (1, 2). Advanced end-stage HF has an unfavorable prognosis, and the ultimate therapeutic option is heart transplantation (HT) (3, 4). In 2021, about 738 HT were performed in China according to data from the China Heart Transplant Registration Network (5). In 2023, the number increased and our center performed 121 HT. The total number of HT reached 1,000 in April 2023. Due to the mismatch between organ supply and demand, there is a significantly higher waitlist than HT surgery rates (6, 7). This leads to longer waiting periods before HT, which may cause disease progression and poor nutritional status (2, 8, 9).

Malnutrition is common in HF, affecting up to 70% of HF patients (10). As HF progresses, it may appear as “cardiac cachexia” in extreme states. In this state, patients would develop protein-calorie malnutrition along with muscle wasting and peripheral edema (11). It leads to a poor life quality and an increase in mortality. However, less severe malnutrition is hard to recognize and the same as its effect on prognosis of HT. An easy and accessible mortality prediction model using nutrition-related indicators may reflect the effect of mild malnutrition on the prognosis of HT and predict mortality risk of HT patients. To our knowledge, there is no such clinical model yet.

Nutritional risk index (NRI) is an easily calculated index incorporating albumin and body size (12). For the past few years, NRI has proven its prognostic utility in HF patients, but there is little data, especially Chinese data, in HT (13–15). In this study, we verified the prognostic value of NRI in HT. Moreover, we used HT patients' data in China to derivate and validate risk prediction models for post-HT surgery death. We aimed at developing and validating a mortality prediction model for HT. This risk stratification approach can be used to identify patients at high risk of death after HT.

## 2 Method

### 2.1 Ethical statement

After donor brain death, all donor hearts were donated to the Red Cross Society in the terms of China's laws. The donor hearts transplanted to recipients were allocated by the China Organ Transplant Response System. The study conformed to the “Declaration of Istanbul on Organ Trafficking and Transplant Tourism” and the national program for deceased organ donation in China (national protocol for China category I) (16). This study was approved by the Ethics Committee of Wuhan Union Hospital. And the requirement of written informed consent was waived by the ethics committee since the study was retrospective. In addition, all clinical data was anonymized and de-identified.

### 2.2 Study population

We used the HT database from our center in which participants were followed up through telephone or outpatient visit. For those who could not attend the telephone interview for physical or cognitive reasons, we performed an interview with their relatives to reduce attrition bias. All participants received orthotopic heart transplantation between 1 January 2015 and 31 December 2020. We excluded people who underwent multiple organ transplantation or re-transplantation and those with missing data. Then we divided patients into the derivation and validation cohorts using a simple randomization method. First, a random number was generated for each participant with random seed 20,191,102. Then, the random numbers were sorted in order from smallest to largest. The first 70% of participants were divided into the derivation cohort; the remaining were placed into the validation cohort (Supplementary Figure S1).

### 2.3 Study outcomes

The primary outcome of the study was defined as all-cause postoperative death. Mortality data were obtained from the China Heart Transplant Registration Network until 26 May 2021, where all deaths of HT are required to be registered by law.

### 2.4 Candidate predictors

We identified nutrition-associated candidate predictors available prior to HT operation through published systematic reviews and univariate Cox proportional hazards regression. All the predictors were retrieved from electronic medical records. Laboratory examinations were conducted within 7 days prior to HT operation. NRI was calculated using the following formula: NRI = [1.519 × serum albumin (in g/dl)] + [41.7 × weight (in kg)/ideal body weight (in kg)] (13). We used the Lorentz formula to calculate ideal body weight (IBW) on the basis of patients' height and gender: IBW = height (in cm)−100−[height (in cm) – 150]/4 for men and IBW = height (in cm)−100−[height (in cm) – 150]/2.5 for women (17).

### 2.5 Model derivation

All nutrition-associated candidate predictors with a significance level of 0.1 in univariate Cox proportional hazards regression were included as potential variables in multivariate Cox proportional hazards regression models in the derivation cohort. To create prediction models that could be more efficiently used, we performed stepwise backward variable selection based on Akaike Information Criterion (AIC) in 1,000 bootstrapped samples with a significance level of 0.05 (18, 19). The bootstrapped samples were the same size as the derivation sample. Then, we fit a reduced model and compared the full prediction model with NRI and the reduced model.

### 2.6 Model performance

The overall goodness-of-fit of the models was compared between models using an AIC indicator. Model discrimination was evaluated through C statistic and integrated discrimination improvement (IDI). As for calibration, calibration curves were drawn graphically. We calculated categorical and continuous net reclassification index to compare the reclassification ability of clinical prediction models (20). For the categorical net reclassification index, the risk threshold was defined as less than 20%, 20% to less than 40%, and 40% or higher.

The area under the ROC curve (AUC) was calculated to validate the discrimination of NRI in overall mortality after HT surgery. Kaplan–Meier (KM) survival analysis was generated to compare survival rate in different groups and differences were examined using log-rank. Statistical significance was considered as a *P*-value of <0.05 (two-sided) for all contrasts. Statistical analysis was conducted using SPSS 27.0.1 and R 4.3.0.

## 3 Results

### 3.1 Characteristics of cohorts

A total of 428 HT patients were included in the study cohort (299 participants in the derivation cohort and 129 in the validation cohort). In the derivation cohort, 240 (80.3%) participants were male with mean (SD) age 48.67 (12.33) years. Most participants (181, 60.5%) were diagnosed with ischemic cardiomyopathy. About 79 (26.4%) underwent cardiac surgery beforehand. The mean (SD) NRI was 100.47 (11.89). By the end of follow up, a total of 66 (22.1%) participants died after HT (Table 1 and Supplementary Table S1).

**Table 1:** Baseline characteristics of derivation and validation cohorts.

Variables	Derivation cohort (*n* = 299)	Validation cohort (*n* = 129)	*P*-value
Recipients			
Gender (male)	240 (80.3%)	102 (79.1%)	0.777
Age (years)	48.67 ± 12.33	46.12 ± 11.99	0.076
BMI (kg/m^2^)	22.88 ± 4.04	23.31 ± 3.75	0.281
Diagnosis			0.096
Ischemia cardiomyopathy	181 (60.5%)	91 (70.5%)	
Non-ischemia cardiomyopathy	69 (23.1%)	17 (13.2%)	
Congenital heart disease	43 (14.4%)	21 (16.3%)	
Other heart disease	6 (2.0%)	0 (0%)	
ABO blood type			0.086
A	107 (35.8%)	38 (29.5%)	
B	72 (24.1%)	43 (33.3%)	
O	95 (31.8%)	43 (33.3%)	
AB	25 (8.4%)	5 (3.9%)	
Hypertension	51 (17.1%)	18 (14.0%)	0.464
Diabetes mellitus	49 (16.4%)	15 (11.6%)	0.184
Hyperlipemia	12 (4.0%)	7 (5.4%)	0.550
Chronic liver disease	23 (7.7%)	9 (7.0%)	0.796
Chronic kidney disease	20 (6.7%)	6 (4.7%)	0.418
History of smoking	119 (39.8%)	58 (45.0%)	0.320
History of alcoholism	69 (23.1%)	35 (27.1%)	0.369
Cardiac surgery history (yes)	79 (26.4%)	32 (24.8%)	0.726
IABP	5 (1.7%)	2 (1.6%)	0.927
ECMO	5 (1.7%)	0 (0%)	0.140
Donors Characteristics			
Donor gender (male)	267 (89.3%)	110 (85.6%)	0.251
Donor age (years)	35.51 ± 11.64	35.04 ± 12.51	0.710
Donor BMI (kg/m^2^)	22.54 ± 3.14	22.71 ± 3.93	0.634
Donor/recipient BMI	1.01 ± 0.20	0.99 ± 0.22	0.441
Donor/recipient age	0.79 ± 0.37	0.81 ± 0.41	0.586
Donor/recipient gender			0.116
Male/male	221 (73.9%)	87 (67.4%)	
Male/female	46 (15.4%)	17 (13.2%)	
Female/male	19 (6.4%)	15 (11.6%)	
Female/female	13 (4.3%)	10 (7.8%)	
Recipient/donor blood-type			0.423
Identical	243 (81.3%)	109 (84.5%)	
Different	56 (18.7%)	20 (15.5%)	
Cause of death			0.173
Brain Injury	186 (64.8%)	66 (53.7%)	
Cerebral hemorrhage	85 (29.6%)	50 (40.7%)	
Brain Tumor	10 (3.5%)	4 (3.3%)	
Others	6 (2.1%)	3 (2.4%)	
Cold ischemia time (min)	333.83 ± 106.69	336.34 ± 114.71	0.827
Aortic crossclamp time (min)	32.05 ± 12.43	33.44 ± 19.90	0.380
Cardiopulmonary bypass time (min)	113.26 ± 37.28	123.67 ± 94.30	0.103
Preoperative Blood Index			
Hb (g/L)	134.60 ± 22.16	134.02 ± 21.27	0.804
RBC (10^12^/L)	4.46 ± 0.72	4.47 ± 0.78	0.911
HCT (%)	40.67 ± 6.22	40.47 ± 6.15	0.763
Bilirubin (µmol/L)	28.27 ± 21.02	28.42 ± 27.63	0.947
ALT (U/L)	72.62 ± 315.98	46.75 ± 85.36	0.361
AST (U/L)	62.92 ± 274.84	38.86 ± 80.07	0.330
SCr (µmol/L)	98.82 ± 43.54	99.24 ± 65.57	0.937
BUN (mmol/L)	8.31 ± 3.97	7.66 ± 3.07	0.097
UA (µmol/L)	503.16 ± 176.06	474.07 ± 153.87	0.104
TC (mmol/L)	3.63 ± 1.00	3.62 ± 0.92	0.910
BNP	5,365.37 ± 6,173.90	4,861.12 ± 5,928.09	0.472
LDL-C (mmol/L)	2.23 ± 0.79	2.20 ± 0.75	0.714
TG (mmol/L)	1.17 ± 0.66	1.23 ± 0.69	0.344
NRI (pg/ml)	100.47 ± 11.89	102.75 ± 10.42	0.060

BMI, body mass index; IABP, intra-aortic balloon pump; ECMO, extracorporeal membrane oxygenation; Hb, hemoglobin; RBC, red blood cell; HCT, hematocrit; ALT, alanine transaminase; AST, aspartate transaminase; SCr, serum creatine; BUN, blood urea nitrogen; UA, uric acid; TC, total cholesterol; BNP, brain natriuretic peptide; LDL-C, low density lipoprotein-cholesterol; TG, triglyceride; NRI, nutritional risk index.

Demographics in the validation cohort were similar to the derivation cohort. And 28 (21.7%) participants died by the end of follow up. Donors' characteristics were also available in the study and there were no statistical differences between the derivation cohort and validation cohort.

### 3.2 Prediction performance of nutritional risk Index

In the derivation cohort, the AUC of NRI for predicting overall postoperative death was 0.613, with a cut-off level of 103.79 (95% CI, 0.542–0.684, *P* = 0.005). Patients in the low NRI group had lower body mass index, hemoglobin, red blood cells, hematocrit, total cholesterol, low density lipoprotein, and lower triglyceride (TG) and higher levels of blood urea nitrogen and brain natriuretic peptide. These patients presented lower prevalence of hypertension and diabetes mellitus and higher prevalence of chronic liver disease (Supplementary Table S2). The result of the K–M survival curve showed that the high NRI group had better overall survival (OS) compared to the low NRI group (*P* < 0.01) (Supplementary Figure S2). The C statistic was 0.59 (95% CI, 0.53–0.66) in the derivation cohort and 0.63 (95% CI, 0.53–0.73) in the validation cohort (Tables 2, 3).

**Table 2:** Mortality after heart transplantation predictors and performance of models in the derivation cohort^a^.

	Models, odds ratio (95% CI)		
	1. Age, NRI, TG, SCr	2. Age, NRI	3. NRI
Predictors			
Age, per year increase	1.04 (1.02–1.07)	1.04 (1.02–1.07)	
NRI, per unit increase	0.98 (0.96–0.99)	0.97 (0.96–0.99)	0.98 (0.96–0.99)
TG, mmol/L			
≥1.2	1 [Reference]		
0.6≤1.2	2.62 (1.26–5.44)		
<0.6	4.99 (2.01–12.39)		
SCr, µmol/L			
<85	1 [Reference]		
85≤130	1.80 (1.02–3.18)		
≥130	2.29 (1.10–4.78)		
Model performance measures			
Akaike information criterion	683.4	696.8	710.6
C statistic	0.72 (0.67–0.78)	0.68 (0.62–0.74)	0.59 (0.53–0.66)
Integrated discrimination improvement, %^b^		6.9 (1.8–15.1)	14.7 (7.4–26.2)
*P*-value		0.005	<0.001
Net reclassification improvement, %			
Continuous		36.9 (17.0–51.6)	46.6 (30.5–64.3)
*P*-value		0.007	<0.001
Categorical^c^		21.2 (−2.8–38.5)	41.8 (9.9–58.8)
*P*-value		0.039	<0.001

NRI, nutritional risk index.

^a^ Of the 299 participants in the derivation cohort, 66 died after heart transplantation.

^b^ Model-2 and -3 were each compared with model-1. The integrated discrimination improvement values and net reclassification improvement values greater than 0 indicated that the four-variable model performed better than other models.

^c^ Risk categories include patients with less than 20%, 20% to less than 40%, and 40% or higher risk of death after heart transplantation.

**Table 3:** Predictive performance of models in the validation cohort^a^.

	Models, odds ratio (95% CI)		
	1. Age, NRI, Scr, TG	2. Age, NRI	3. NRI
Akaike information criterion	252	253	258.1
C statistic	0.71 (0.62–0.81)	0.67 (0.57–0.77)	0.63 (0.53–0.73)
Integrated discrimination improvement, %^b^		4.3 (−0.4–15.3)	13.2 (3.6–31.7)
*P*-value		0.085	0.003
Net reclassification improvement, %			
Continuous		25.5 (−8.2–48.8)	40.0 (10.3–65.0)
*P*-value		0.142	0.017
Categorical^c^		20.6 (−9.1–56.7)	60.7 (9.0–100.5)
*P*-value		0.22	0.008

NRI, nutritional risk index.

^a^ Of the 129 participants in the validation cohort, 28 died after heart transplantation.

^b^ Model-2 and -3 were each compared with model-1. The integrated discrimination improvement values and net reclassification improvement values greater than 0 indicated that the four-variable model performed better than other models.

^c^ Risk categories include patients with less than 20%, 20% to less than 40%, and 40% or higher risk of death after heart transplantation.

### 3.3 Nutrition-associated prediction model derivation

In the bootstrapped samples of the derivation cohort, multivariable Cox proportional hazards regression analysis showed that older age, lower NRI values, and higher serum creatinine (SCr) and TG values were relevant to a high risk of death after HT. After this, the four-variable model (model-1) was finally developed. The odds ratio of multivariable Cox proportional hazards regression for variables in the model can be seen in Table 2.

### 3.4 Prediction model performance in the derivation and validation cohort

The AUC of the four-variable model (model-1) for predicting overall postoperative death was 0.755 in the derivation cohort. (Supplementary Figure S3A) Compared with other models, model-1 had the highest C statistic and lowest AIC both in the derivation and validation cohorts. The C statistic of model-1 was 0.72 (95% CI, 0.67–0.78) in the derivation cohort and 0.71 (95% CI, 0.62–0.81) in the validation cohort. Discrimination based on IDI significantly improved in the four-variable model compared with model-2 without SCr and TG (6.9%; 95% CI, 1.8%–15.1%; *P* < 0.01) and with model-3, which only included variable NRI (14.7%; 95% CI, 7.4%–26.2%; *P* < 0.001) in the derivation cohort (Tables 2, 3). A similar IDI improvement was also observed in the validation cohort. Both continuous and categorical net reclassification index improved in the four-variable model compared with other models in the derivation and validation cohorts.

**Figure 1:**
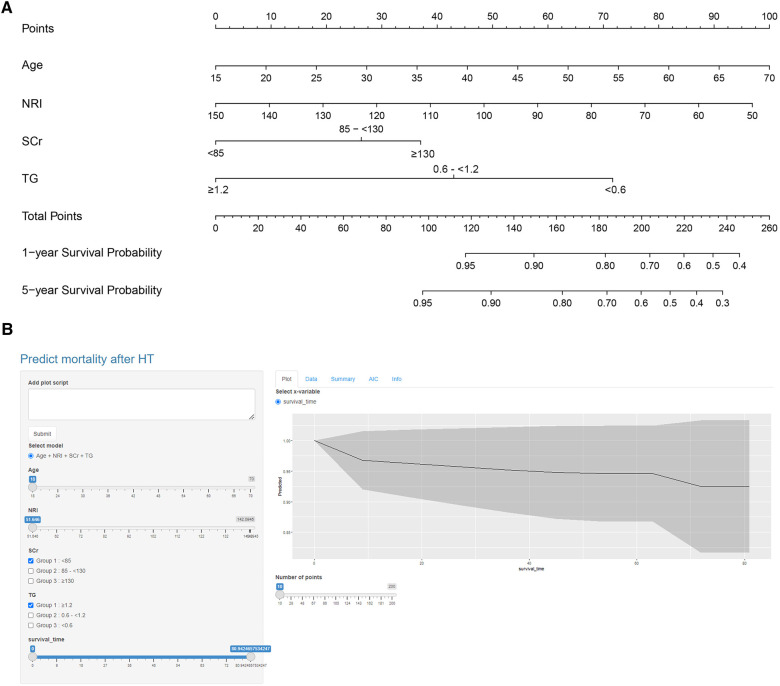
Nomogram of the four-variable model. (**A**) Nomogram was constructed based on the derivation cohort. The range of indicators in the nomogram was shown as follows: Age, 18–70 years old; NRI, 51.65–142.09; SCr, 36.1–531.5 µmol/L; TG, 0.25–4.79 mmol/L. (**B**) The dynamic nomogram is available online (https://docqianofwuhanunionhospital.shinyapps.io/MortalityPredictionAfterHeartTransplantation/).

As the calibration curves show, the four-variable model was better calibrated than other models in the derivation cohort and was the same in the validation cohort (Supplementary Figure S4). Based on the predicted risk of 5-year post-HT death calculated through the four-variable model, 155 patients (51.8%) in the derivation cohort had less than 20%; 102 (34.1%) 20% to less than 40%; and 42 (14.0%) 40% or more risk of death (Supplementary Table S3). The K–M survival curve analysis demonstrated that participants in the group with a predicted risk of 5-year post-HT death less than 20% had better OS compared to that of 20% to less than 40% risk of postoperative death and 40% or more risk of death (*P* < 0.0001) (Supplementary Figure S3B). Then we presented the four-variable model as a nomogram (Figure 1A) and made it freely available online to help clinicians to calculate the risk of post-HT death (Figure 1B) (https://docqianofwuhanunionhospital.shinyapps.io/MortalityPredictionAfterHeartTransplantation/).

## 4 Discussion

In this study, we first investigated the prediction efficiency of NRI on post-HT surgery death. The results showed that patients with higher NRI had lower OS post operation. Patients in the low NRI group had a higher prevalence of liver disease. As reported, malnutrition occurs in more than 50% of patients with chronic liver disease. Both adipose tissue and muscle tissue can be depleted; female patients more frequently develop a depletion in fat deposits while males more rapidly lose muscle tissue (21). Patients with low NRI had lower hemoglobin, fewer red blood cells, and higher blood urea nitrogen. The reason for that may be these patients had more severe primary disease, which would affect the nutritional status of the body.

Then, we developed a four-variable mortality prediction model using nutrition-related indicators. It can be used to estimate mortality risk 1-year and 5-year post-HT operation and is available online. This model included variables of age, NRI, SCr, and TG, which can be obtained readily in clinical practice, and showed the best performance for predicting postoperative death of HF patients in derivation and validation cohorts than reduced models based on age and NRI, or based on NRI alone. In clinical practice, it can be used easily to estimate individualized risk of death post HT operation. Death risk stratification according to this model could help guide prognostic assessment and medical care after admission.

As the results of the model discrimination and calibration showed, there was no deterioration in the validation cohort, which means the four-variable nutrition-associated mortality prediction model was not overfit. Generally speaking, a C statistic higher than 0.70 is a criterion to determine whether models are useful in clinical use (22). In this four-variable model, the C statistic was 0.72 (95% CI, 0.67–0.78) in the derivation cohort and 0.71 (95% CI, 0.62–0.81) in the validation cohort. Since both C statistics were higher than 0.70, this model was considered as having significance in clinical decision making. Compared to the reduced model and variable NRI alone, net reclassification improvement of the full model demonstrated that the four-variable nutrition-associated mortality prediction model could improve accuracy of predicting post-HT death and risk stratification of death.

Singh et al. reported a risk prediction model to predict in-hospital mortality post HT operation using six recipient variables in 2012 (23). The model was derived in HT participants in the United States and validated internally through bootstrapping method and externally in patients receiving HT from July 2009 to October 2010. The C statistics were 0.72 in the derivation cohort, 0.73 in the internal validation cohort, and 0.68 in the external validation cohort. In addition, Weiss et al. developed a risk score using 12 recipient variables in US recipients from 1997 to 2008. This score could be used to predict 1-year mortality post HT (C statistic, 0.65) (24). Both of these studies focused on early mortality after HT. The mortality risk prediction model using nutrition-related indicators in this study differs importantly by its focus on post-operation 5-year mortality and using a more recent China cohort (2015–2020). There is no other prediction model using Chinese HT data to our knowledge. Besides, the model in this study focused on the malnutrition effect on prognosis of patients who received HT. Hence, all the candidates and the ultimate variables of this model were nutrition-associated indexes.

The mortality prediction model developed in this study may have implications for clinical care and decision making. Obtaining individualized risk of death after HT may help inform decisions about pursuing a course of treatment. Clinicians could use this model to identify patients at high risk of postoperative death before HT surgery, which would help with targeted preventative therapy to reduce the mortality risk. Also, being able to identify patients at high risk of postoperative mortality before HT may allow for better planning of resource allocation. In clinical practice, it is quite challenging for clinicians to determine the therapy of HT, especially in complex HF patients, as there are alternative therapies like implantable ventricular assist devices (25, 26). The mortality risk prediction model may be useful in assessing whether patients could benefit from a transplant.

## 5 Limitations

This study has some limitations. First, several nutrition-associated variables like muscle mass, weight loss within 1 month, triceps' skinfold thickness, and so on were not included in this study since this was a retrospective study and such variables were not attainable in the HT database. Second, participants with retransplant were excluded from this study, so the prediction model may not be appropriate for those patients. Due to the complex condition and extra risk of retransplant patients, a more specific study should be performed for those patients. Finally, the model was developed using HT data from a single center in China and we did not perform external validation for this model, which may influence the efficacy of the model. This work should be verified in the future.

## 6 Conclusion

A multivariable prediction model using variables of age, NRI, SCr, and TG was developed in this study. It was able to predict mortality after HT operation and is available online for use. The utility of this prediction model in clinical practice requires further investigation.

## Data Availability

The original contributions presented in the study are included in the article/**Supplementary Material**, further inquiries can be directed to the corresponding authors.

## References

[1] GBD 2017 Disease and Injury Incidence and Prevalence Collaborators. Global, regional, and national incidence, prevalence, and years lived with disability for 354 diseases and injuries for 195 countries and territories, 1990–2017: a systematic analysis for the global burden of disease study 2017. Lancet. (2018) 392(10159):1789–858. 10.1016/S0140-6736(18)32279-7 30496104 PMC6227754

[2] Ma LY Chen WW Gao RL Liu LS Zhu ML Wang YJ China Cardiovascular diseases report 2018: an updated summary. J Geriatr Cardiol. (2020) 17(1):1–8. 10.11909/j.issn.1671-5411.2020.01.001 32133031 PMC7008101

[3] Yusen RD Edwards LB Dipchand AI Goldfarb SB Kucheryavaya AY Levvey BJ The registry of the international society for heart and lung transplantation: thirty-third adult lung and heart-lung transplant report-2016; focus theme: primary diagnostic indications for transplant. J Heart Lung Transplant. (2016) 35(10):1170–84. 10.1016/j.healun.2016.09.001 27772669

[4] McDonagh TA Metra M Adamo M Gardner RS Baumbach A Böhm M 2021 ESC guidelines for the diagnosis and treatment of acute and chronic heart failure. Eur Heart J. (2021) 42(36):3599–726. 10.1093/eurheartj/ehab368 34447992

[5] Wang Z Ma L Liu M Fan J Hu S, Writing Committee of the Report on Cardiovascular Health and Diseases in China. Summary of the 2022 report on cardiovascular health and diseases in China. Chin Med J. (2023) 136(24):2899–908. 10.1097/CM9.0000000000002927 38018129 PMC10752444

[6] Manara A Shemie SD Large S Healey A Baker A Badiwala M Maintaining the permanence principle for death during in situ normothermic regional perfusion for donation after circulatory death organ recovery: a United Kingdom and Canadian proposal. Am J Transplant. (2020) 20(8):2017–25. 10.1111/ajt.15775 31922653 PMC7540256

[7] Crespo-Leiro MG Costanzo MR Gustafsson F Khush KK Macdonald PS Potena L Heart transplantation: focus on donor recovery strategies, left ventricular assist devices, and novel therapies. Eur Heart J. (2022) 43(23):2237–46. 10.1093/eurheartj/ehac204 35441654

[8] Singhal AK Abrams JD Mohara J Hasz RD Nathan HM Fisher CA Potential suitability for transplantation of hearts from human non-heart-beating donors: data review from the gift of life donor program. J Heart Lung Transplant. (2005) 24(10):1657–64. 10.1016/j.healun.2004.11.043 16210144

[9] Taylor DO Stehlik J Edwards LB Aurora P Christie JD Dobbels F Registry of the international society for heart and lung transplantation: twenty-sixth official adult heart transplant report-2009. J Heart Lung Transplant. (2009) 28(10):1007–22. 10.1016/j.healun.2009.08.014 19782283

[10] Sze S Pellicori P Kazmi S Rigby A Cleland JGF Wong K Prevalence and prognostic significance of malnutrition using 3 scoring systems among outpatients with heart failure: a comparison with body mass index. JACC Heart Fail. (2018) 6(6):476–86. 10.1016/j.jchf.2018.02.018 29753673

[11] Grossniklaus DA O'Brien MC Clark PC Dunbar SB. Nutrient intake in heart failure patients. J Cardiovasc Nurs. (2008) 23(4):357–63. 10.1097/01.JCN.0000317433.52210.e0 18596500 PMC4264831

[12] Yoshihisa A Kanno Y Watanabe S Yokokawa T Abe S Miyata M Impact of nutritional indices on mortality in patients with heart failure. Open Heart. (2018) 5(1):e000730. 10.1136/openhrt-2017-000730 29344381 PMC5761292

[13] Adejumo OL Koelling TM Hummel SL. Nutritional risk index predicts mortality in hospitalized advanced heart failure patients. J Heart Lung Transplant. (2015) 34(11):1385–9. 10.1016/j.healun.2015.05.027 26250966 PMC4619156

[14] Krishnan A Bigelow B Hsu S Gilotra NA Sharma K Choi CW Decreased nutritional risk index is associated with mortality after heart transplantation. Clin Transplant. (2021) 35(5):e14253. 10.1111/ctr.14253 33576056

[15] Almutawa DA Almuammar M Elshafie MM Aljuraiban GS Alnafisah A Abulmeaty MMA. Survival and nutritional status of male and female heart transplant patients based on the nutritional risk index. Nutrients. (2020) 12(12):3868. 10.3390/nu12123868 33348880 PMC7766250

[16] International Summit on Transplant Tourism and Organ Trafficking. The declaration of Istanbul on organ trafficking and transplant tourism. Clin J Am Soc Nephrol. (2008) 3(5):1227–31. 10.2215/CJN.03320708 18701611 PMC4571160

[17] Lee M Lim JS Kim Y Lee JH Kim CH Lee SH Association between geriatric nutritional risk index and post-stroke cognitive outcomes. Nutrients. (2021) 13(6):1776. 10.3390/nu13061776 34070955 PMC8224551

[18] James MT Pannu N Hemmelgarn BR Austin PC Tan Z McArthur E Derivation and external validation of prediction models for advanced chronic kidney disease following acute kidney injury. JAMA. (2017) 318(18):1787–97. 10.1001/jama.2017.16326 29136443 PMC5820711

[19] Liu Q Charleston MA Richards SA Holland BR. Performance of akaike information criterion and Bayesian information criterion in selecting partition models and mixture models. Syst Biol. (2023) 72(1):92–105. 10.1093/sysbio/syac081 36575813 PMC10198649

[20] Cook NR Ridker PM. Advances in measuring the effect of individual predictors of cardiovascular risk: the role of reclassification measures. Ann Intern Med. (2009) 150(11):795–802. 10.7326/0003-4819-150-11-200906020-00007 19487714 PMC2782591

[21] European Association for the Study of the Liver. Electronic address: easloffice@easloffice.eu, & European Association for the Study of the Liver. EASL clinical practice guidelines on nutrition in chronic liver disease. J Hepatol. (2019) 70(1):172–93. 10.1016/j.jhep.2018.06.024 30144956 PMC6657019

[22] Hosmer DW Lemeshow S. Applied Logistic Regression. 2nd ed. New York, NY: John Wiley & Sons (2000).

[23] Singh TP Almond CS Semigran MJ Piercey G Gauvreau K. Risk prediction for early in-hospital mortality following heart transplantation in the United States. Circ Heart Fail. (2012) 5(2):259–66. 10.1161/CIRCHEARTFAILURE.111.965996 22308287

[24] Weiss ES Allen JG Arnaoutakis GJ George TJ Russell SD Shah AS Creation of a quantitative recipient risk index for mortality prediction after cardiac transplantation (IMPACT). Ann Thorac Surg. (2011) 92:914–21. 10.1016/j.athoracsur.2011.04.030 21871277

[25] Brown CR Khurshan F Chen Z Groeneveld PW McCarthy F Acker M Optimal timing for heart transplantation in patients bridged with left ventricular assist devices: is timing of the essence? J Thorac Cardiovasc Surg. (2019) 157(6):2315–24.e4. 10.1016/j.jtcvs.2018.12.118 30955956

[26] Macera F Occhi L Masciocco G Varrenti M Frigerio M. A new life: motherhood after heart transplantation. A single-center experience and review of literature. Transplantation. (2018) 102(9):1538–44. 10.1097/TP.0000000000002281 29762460

