# Supplementary material for: Development and validation of mortality prediction models for heart transplantation using nutrition-related indicators: a single-center study from China
Source: Front Cardiovasc Med. 2024 Feb 26;11:1346202. doi: 10.3389/fcvm.2024.1346202 (PMC10926190; doi:10.3389/fcvm.2024.1346202)
Supplement: Supplementary file 1 [file Datasheet1.docx]

eTable 1. Predictors of mortality after heart transplantation and their mean value or frequency in the derivation and validation cohorts

| Predictors | Derivation Cohort | | Validation Cohort | |
| --- | --- | --- | --- | --- |
|  | Full cohort (n = 299) | Death (n = 66) | Full cohort (n = 129) | Death (n = 28) |
| Receptor age | 48.67 ± 12.33 | 53.41 ± 10.69 | 46.12 ± 11.99 | 51.04 ± 12.24 |
| NRI | 100.47 ± 11.89 | 96.75 ± 11.30 | 102.75 ± 10.42 | 98.82 ± 9.39 |
| SCr |  |  |  |  |
| < 85 | 122 (40.8%) | 19 (28.8%) | 58 (45.0%) | 14 (50.0%) |
| 85 - <130 | 140 (46.8%) | 24 (36.4%) | 58 (45.0%) | 12 (42.9%) |
| ≥ 130 | 37 (12.4%) | 13 (19.7%) | 13 (10.1%) | 2 (7.1%) |
| TG |  |  |  |  |
| ≥ 1.2 | 96 (32.1%) | 9 (13.6%) | 50 (38.8%) | 8 (28.6%) |
| 0.6 - < 1.2 | 175 (58.5%) | 47 (71.2%) | 75 (58.1%) | 19 (67.9%) |
| < 0.6 | 28 (9.4%) | 10 (15.2%) | 4 (3.1%) | 1 (3.6%) |

Abbreviations: SCr, serum creatine; TG, triglyceride; NRI, nutritional risk index.

eTable 2. Baseline Characteristics of low and high NRI groups

| Variables | Low NRI group (n = 179) | High NRI group (n = 120) | *P* value |
| --- | --- | --- | --- |
| **Recipients** |  |  |  |
| Gender (male) | 140 (78.2%) | 100 (83.3%) | 0.275 |
| Age (years) | 47.78 ± 13.47 | 49.98 ± 10.32 | 0.130 |
| BMI (kg/m^2^) | 20.98 ± 3.04 | 25.7 ± 3.67 | < 0.001 |
| Diagnosis |  |  | 0.016 |
| Ischemia cardiomyopathy | 31 (17.3%) | 38 (31.7%) |  |
| Non-ischemia cardiomyopathy | 111 (62.0%) | 70 (58.3%) |  |
| Congenital heart disease | 30 (16.8%) | 12 (10.0%) |  |
| Other heart disease | 7 (4.0%) | 0 (0.0%) |  |
| ABO blood type |  |  | 0.424 |
| A | 59 (33.0%) | 48 (40.0%) |  |
| B | 43 (24.0%) | 29 (24.2%) |  |
| O | 63 (35.2%) | 32 (26.7%) |  |
| AB | 14 (7.8%) | 11 (9.2%) |  |
| Hypertension | 23 (14.1%) | 28 (25.2%) | 0.020 |
| Diabetes mellitus | 20 (14.0%) | 29 (30.2%) | 0.002 |
| Hyperlipemia | 7 (5.2%) | 5 (5.3%) | 0.975 |
| Chronic liver disease | 19 (10.6%) | 4 (3.3%) | 0.021 |
| Chronic kidney disease | 13 (7.3%) | 7 (5.8%) | 0.628 |
| History of smoking | 69 (38.5%) | 50 (41.7%) | 0.589 |
| History of alcoholism | 41 (22.9%) | 28 (23.3%) | 0.931 |
| Cardiac surgery history (yes) | 50 (27.9%) | 29 (24.2%) | 0.469 |
| IABP | 4 (2.2%) | 1 (0.8%) | 0.354 |
| ECMO | 3 (1.7%) | 2 (1.7%) | 0.995 |

eTable 2. Baseline Characteristics of low and high NRI groups (continued)

| Variables | Low NRI group (n = 179) | High NRI group (n = 120) | *P* value |
| --- | --- | --- | --- |
| **Donors Characteristics** |  |  |  |
| Donor gender (male) | 158 (88.3%) | 109 (90.8%) | 0.482 |
| Donor age (years) | 34.94 ± 11.86 | 36.35 ± 11.31 |  |
| Donor BMI (kg/m^2^) | 22.36 ± 3.02 | 22.82 ± 3.32 |  |
| Donor/recipient BMI | 1.08 ± 0.19 | 0.90 ± 0.16 |  |
| Donor/recipient age | 0.81 ± 0.41 | 0.76 ± 0.30 |  |
| Donor/recipient gender |  |  | 0.572 |
| Male/male | 129 (72.1%) | 92 (76.7%) |  |
| Male/female | 29 (16.2%) | 17 (14.2%) |  |
| Female/male | 11 (6.1%) | 8 (6.7%) |  |
| Female/female | 10 (5.6%) | 3 (2.5%) |  |
| Recipient/donor blood-type |  |  | 0.454 |
| Identical | 143 (79.9%) | 100 (83.3%) |  |
| Different | 36 (20.1%) | 20 (16.7%) |  |
| Cause of death |  |  | 0.525 |
| Brain Injury | 113 (63.1%) | 73 (60.8%) |  |
| Cerebral hemorrhage | 48 (26.8%) | 37 (30.8%) |  |
| Brain Tumor | 5 (2.8%) | 5 (4.2%) |  |
| Others | 13 (7.3%) | 5 (4.2%) |  |
| Cold ischemia time (min) | 334.92 ± 111.31 | 332.20 ± 100.12 |  |
| Aortic crossclamp time (min) | 32.47 ± 14.82 | 31.41 ± 7.59 |  |
| Cardiopulmonary bypass time (min) | 116.62 ± 41.90 | 108.26 ± 28.51 |  |

eTable 2. Baseline Characteristics of low and high NRI groups (continued)

| Variables | Low NRI group (n = 179) | High NRI group (n = 120) | *P* value |
| --- | --- | --- | --- |
| **Preoperative Blood Index** |  |  |  |
| Hb (g/L) | 130.83 ± 23.36 | 140.22 ± 18.98 | < 0.001 |
| RBC (10^12^/L) | 4.33 ± 0.76 | 4.66 ± 0.60 | < 0.001 |
| HCT (%) | 39.55 ± 6.35 | 42.33 ± 5.66 | < 0.001 |
| Bilirubin (μmol/L) | 29.92 ± 21.96 | 26.25 ± 20.28 | 0.146 |
| ALT (U/L) | 64.27 ± 169.45 | 85.08 ± 454.78 | 0.577 |
| AST (U/L) | 62.91 ± 243.63 | 62.94 ± 316.82 | 0.999 |
| SCr (μmol/L) | 101.50 ± 50.28 | 94.82 ± 30.64 | 0.193 |
| BUN (mmol/L) | 8.85 ± 4.34 | 7.50 ± 3.19 | 0.004 |
| UA (μmol/L) | 496.08 ± 186.30 | 513.73 ± 159.75 | 0.397 |
| TC (mmol/L) | 3.50 ± 0.92 | 3.82 ± 1.09 | 0.007 |
| BNP | 6834.57 ± 7248.62 | 3353.50 ± 3405.03 | < 0.001 |
| LDL-C (mmol/L) | 2.15 ± 0.75 | 2.35 ± 0.83 | 0.038 |
| TG (mmol/L) | 1.02 ± 0.50 | 1.39 ± 0.79 | < 0.001 |

eTable 3. Frequency, and observed risk of risk categories for 3 models for mortality after HT in the derivation cohort

| Independent Variables | Predicted Risk Category | Sample Size (%) in Each Predicted Risk Category (%) | Postoperative Death, N (%) |
| --- | --- | --- | --- |
| Age, NRI, SCr, TG | < 20% | 155 (51.8) | 14 (9.0) |
|  | 20% - < 40% | 102 (34.1) | 32 (31.4) |
|  | ≥ 40% | 42 (14.0) | 20 (47.6) |
| Age, NRI | < 20% | 137 (45.8) | 15 (10.9) |
|  | 20% - < 40% | 145 (48.5) | 42 (29.0) |
|  | ≥ 40% | 17 (5.7) | 9 (52.9) |
| NRI | < 20% | 106 (35.5) | 14 (13.2) |
|  | 20% - < 40% | 186 (62.2) | 50 (26.9) |
|  | ≥ 40% | 7 (23.4) | 2 (28.6) |

There was a total of N = 299 patients in the derivation cohort, with 66 death after HT events in the derivation cohort.

Abbreviations: SCr, serum creatine; TG, triglyceride; NRI, nutritional risk index.





eFigure 1. Formation of the Derivation and Validation Cohorts of this Study


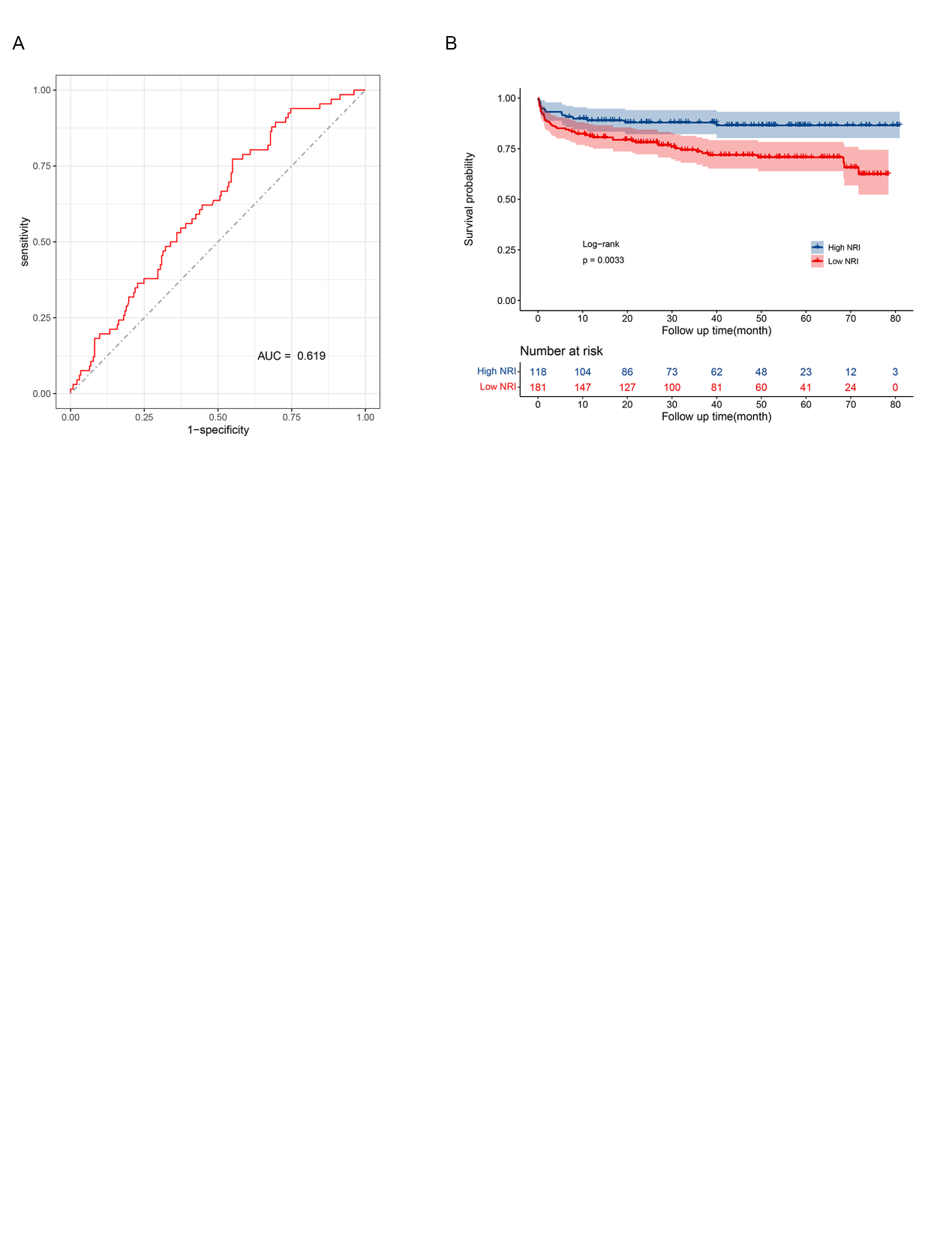


eFigure2. ROC and K-M Survival Curves of NRI in the Derivation Cohort. A. The AUC of NRI for predicting overall postoperative death was 0.613, with a cut-off level of 103.79. B. High NRI group mean patients with NRI equal to and higher than 103.79 and low NRI group mean NRI lower than 103.79. High NRI group had better overall survival compared to the low NRI group.


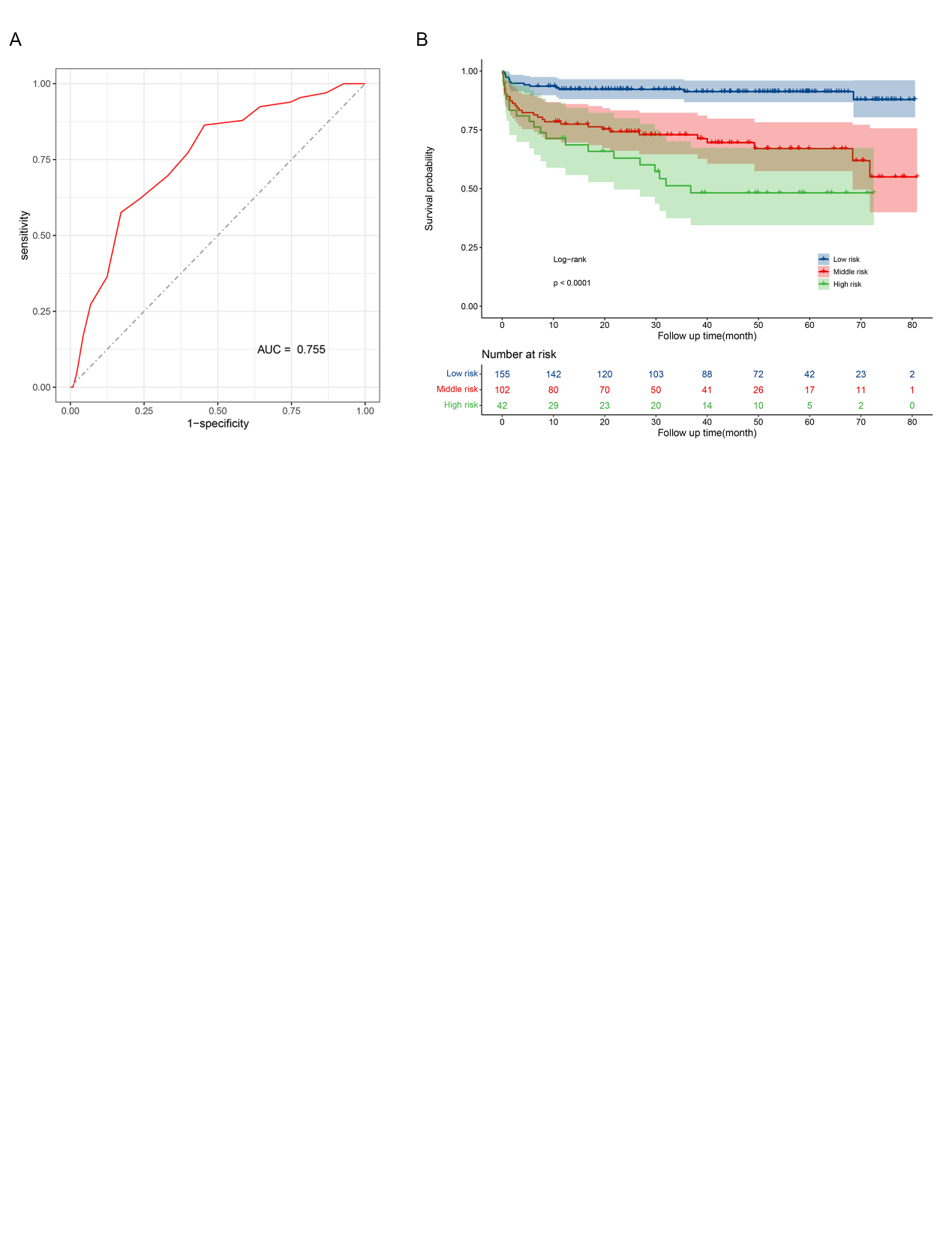


eFigure 3. ROC and K-M Survival Curves of the 4-variable model in the Derivation Cohort. A. The AUC of the 4-variable model for predicting overall postoperative death was 0.755. B. Low risk mean patients with predicted risk of 5-year post HT death less than 20%, middle mean 20% to less than 40% risk of postoperative death and high risk mean 40% or more risk of death. Low risk patients had highest overall survival compared to other two groups.


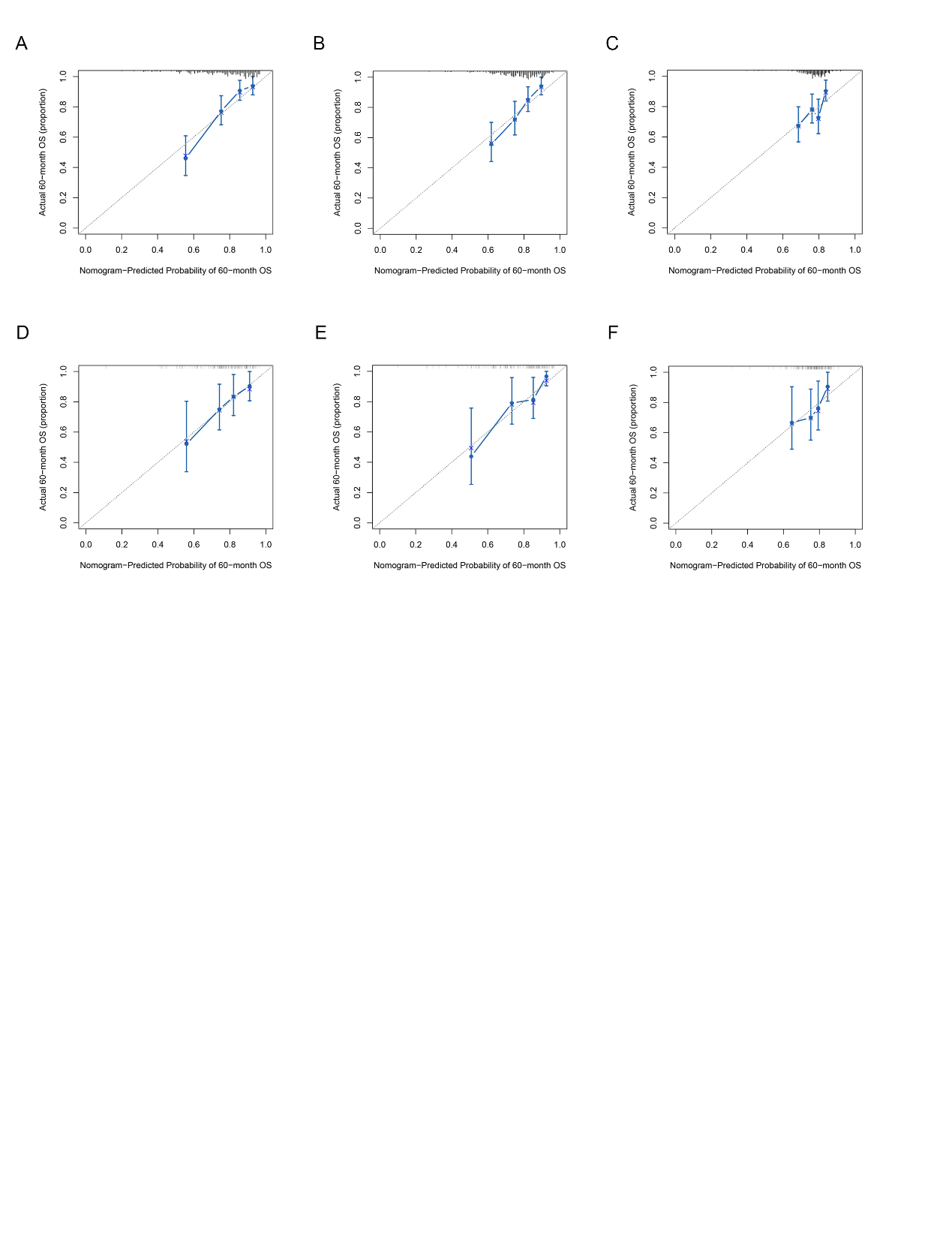


eFigure 4. Calibration Curves of Models in the Derivation and Validation cohorts. A-C. Calibration curves of model-1 to model-3 in the derivation cohort. D-F. Calibration curves of model-1 to model-3 in the validation cohort.
